# Breastfeeding Duration Is Associated with the Risk of Tooth Loss, Chewing Difficulty, and Undernutrition among Older Korean Women: Results of the Korea National Health and Nutrition Examination Survey (KNHANES) 2013–2015

**DOI:** 10.3390/nu15245024

**Published:** 2023-12-07

**Authors:** Ye Rang Jo, Yoo Kyoung Park, Hee-Sook Lim

**Affiliations:** 1 Department of Medical Nutrition, Graduate School of East-West Medical Science, Kyung Hee University, Yongin 17104, Republic of Korea; joyerang@khu.ac.kr; 2 Department of Gerontology, AgeTech-Service Convergence Major, Graduate School of East-West Medical Science, Kyung Hee University, Yongin 17104, Republic of Korea

**Keywords:** older Korean women, breastfeeding duration, tooth loss, chewing difficulty, undernutrition

## Abstract

We investigated whether older Korean women with prolonged breastfeeding duration have an increased risk of tooth loss, chewing difficulty, or undernutrition, as well as whether tooth loss and chewing difficulty mediate the association between breastfeeding duration and undernutrition risk. This study included 1666 women aged ≥65 years from the 2013–2015 Korea National Health and Nutrition Examination Survey who breastfed after delivery. The number of teeth and chewing ability were investigated based on the status of individual teeth and a self-report questionnaire, respectively. Dietary intake was estimated using the 24 h recall method. Compared with women who breastfed for 1–18 months, the odds ratios for tooth loss were 1.16 (95% confidence interval [CI] = 0.69–1.94), 1.79 (95% CI = 1.08–2.94), and 1.86 (95% CI = 1.16–2.97) among women who breastfed for 19–36, 37–72, and ≥73 months, respectively (*p* for trend = 0.004). Similar results were obtained for chewing difficulty and undernutrition. Furthermore, tooth loss and chewing difficulty partially mediated the association between breastfeeding duration and undernutrition risk. In conclusion, older Korean women who breastfed for longer periods are more likely to experience tooth loss, chewing difficulty, and undernutrition, which are particularly severe among women who breastfed for ≥37 months. The association between breastfeeding duration and undernutrition risk is mediated by tooth loss and chewing difficulty.

## 1. Introduction

Breastfeeding yields health benefits for mothers such as a reduced risk of chronic diseases, including type 2 diabetes, hypertension, breast cancer, and ovarian cancer [1]. Conversely, breastfeeding affects maternal bone metabolism by utilizing approximately 300–400 mg of calcium daily for breast milk production. Previous studies show that bone loss can occur during breastfeeding [2,3]. This phenomenon can be explained by the rapid bone turnover associated with breastfeeding [4,5,6]. Breastfeeding leads to the secretion of parathyroid hormone-related protein (PTHrP) and estrogen deficiency, which further result in differentiation of osteoclast precursors [6,7,8,9]. Notably, breastfeeding-induced bone loss is temporary, with complete recovery achieved within 6–12 months after stopping breastfeeding [6,10]. Osteoclast apoptosis occurs after breastfeeding is stopped and the PTHrP and estrogen levels return to normal [6,11]. A systematic review of prospective human studies demonstrated temporary breastfeeding-induced bone loss with complete recovery or a tendency toward recovery. The included studies primarily measured bone density at specific sites, such as the lumbar spine, hip, and forearm, as well as the entire body [12].

Alveolar bone surrounds the tooth roots and forms the tooth sockets within the jawbone that hold teeth in place [13,14]. Previous studies have suggested that breastfeeding is associated with reduced alveolar bone in rats [15,16]. Jawbone osteoclasts express higher levels of anti-apoptotic genes, including Bcl-2 and Bcl-xL, compared to long bone osteoclasts [17]. This suggests that alveolar bone loss caused by breastfeeding could be less likely to be fully recovered. Breastfeeding women might be more susceptible to tooth loss, which is supported by a previous study of Korean postmenopausal women [18]. The study revealed negative associations of the total breastfeeding duration and average breastfeeding duration per infant with the number of natural teeth. On the other hand, one of the essential functions of teeth is to chew food [19]. Individuals with fewer teeth or impaired chewing ability are likely to alter their food choices based on their oral status, potentially leading to negative effects on their nutritional status [20,21]. 

The upper limit of the breastfeeding duration is not defined [22]. However, prolonged breastfeeding might pose unique challenges for older Korean women, as many of them practice unusually prolonged breastfeeding. Conversely, North American mothers typically follow a social norm that encourages weaning at 6 months following delivery [23], with a breastfeeding rate of 24.7% in the United States in 1971 [24]. On the other hand, in Korea the tradition is to breastfeed for 3 years [25], and 99.7% of Korean infants were breastfed in 1970 [26]. We anticipate that prolonged breastfeeding can lead to a higher risk for tooth loss, chewing difficulty, and undernutrition among older Korean women. However, only one study [18] examined the link between breastfeeding duration and number of teeth. And the study included Korean postmenopausal women and did not address the link between breastfeeding duration, chewing ability, and nutritional status. Here, we investigated this question and determined whether tooth loss and chewing difficulty mediate the association between breastfeeding duration and undernutrition risk, utilizing nationally representative data. 

## 2. Materials and Methods

### 2.1. Study Participants

**Figure 1:**
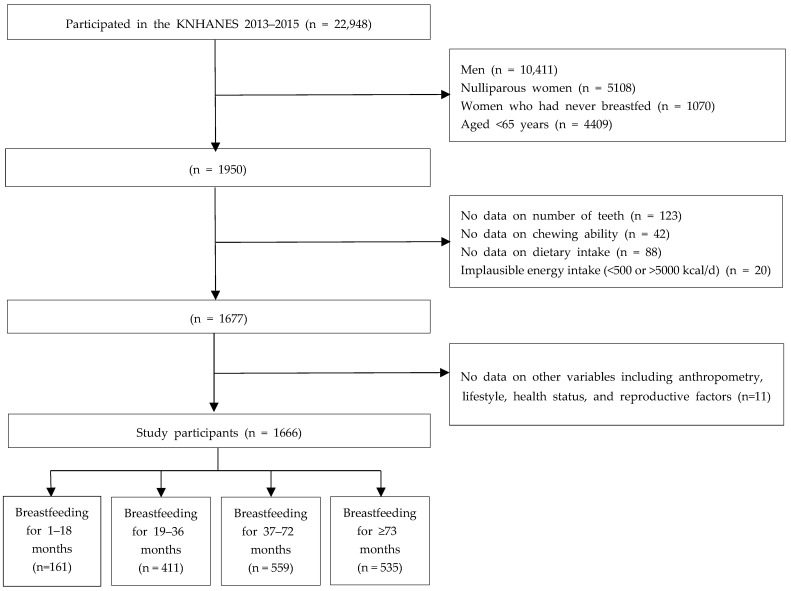
Flowchart of study participants. KNHANES, Korea National Health and Nutrition Examination Survey.

This cross-sectional study was conducted using data from the 2013–2015 Korea National Health and Nutrition Examination Survey (KNHANES). KNHANES is a survey of community-dwelling South Korean civilians and comprises health interviews, health examinations, and nutrition assessments. The survey is conducted by Korea Centers for Disease Control and Prevention (KCDC) every year [27]. The Institutional Review Board (IRB) of the KCDC approved the 2013 (IRB approval number: 2013-07CON-03-4C) and 2014 (IRB approval number: 2013-12EXP-03-5C) surveys. The 2015 survey was posted as research conducted by the nation for public welfare and thus was exempt from review by the IRB at the KCDC per the Bioethics and Safety Act. This is described in the Guidebook for KNHANES database [28]. 1950 women aged ≥65 years who had a history of breastfeeding following delivery were included in KNHANES. We excluded 284 individuals with missing data or implausible energy intake (<500 or >5000 kcal/d) [29]. Finally, this study included 1666 individuals. The breastfeeding duration was determined by participant recall and defined as the total lifetime duration of breastfeeding. Those who answered “yes” to the question “Have you ever breastfed for at least one month?” were considered to have breastfeeding experience. They were asked about the total breastfeeding duration. Breastfeeding duration was categorized into four groups based on previous study examining the association between breastfeeding duration and sarcopenia in elderly Korean women: 1–18, 19–36, 37–72, and ≥73 months (*n* = 161, =411, =559, and =535, respectively) [30]. Figure 1 presents a flowchart of participant selection. 

### 2.2. Anthropometry, Lifestyle, Health Status, and Reproductive Factors 

Trained examiners measured the height and weight while participants were wearing light indoor clothing and no shoes, ensuring precision to the nearest 0.1 cm and 0.1 kg, respectively. Body mass index (BMI) was calculated by dividing weight (kg) by height squared (m^2^). Smoking was defined as having consumed more than five packs (100 cigarettes) over the entire lifetime [31]. Drinking was defined as having consumed alcohol at least once a month during the past year [31]. Regular walking was defined as walking ≥30 min at least five times per week [32]. The KNHANES also recorded whether individuals brush their teeth before and after breakfast, before and after lunch, before and after dinner, after snacks, and before going to bed. Using this information, the daily frequency of tooth brushing was categorized as ≤1, 2 or ≥3, with values ranging between 0 and 8. We recorded the presence of chronic diseases, including hypertension, diabetes, dyslipidemia, stroke/myocardial infarction/angina pectoris, and cancer, based on diagnoses made by a doctor. Additionally, we categorized the number of chronic diseases as 0, 1, or ≥2. And the reproductive factors were determined by participant recall. Number of pregnancies was identified for those who answered “yes” to the question “Have you ever been pregnant (including current pregnancy, spontaneous abortion, artificial abortion, ectopic pregnancy, and etc.)?”. Age at last birth was investigated for those who answered “yes” to the question “Have you ever given birth (including normal birth, preterm birth, and etc.)?”. We calculated time since last birth by subtracting age at last birth from current age. Breastfeeding duration was obtained as described above. 

### 2.3. Number of Teeth and Chewing Ability 

To estimate the number of teeth, we used the status of individual teeth. Dentists categorized the status of individual teeth as 0, 1, 3, 4, 5, 6, 7, 8, and 9 corresponding to healthy, caries, caries-experienced treatment, loss of caries-experienced, loss of caries-non-experienced, full-color, caries-non-experienced treatment, unerupted, and non-recordable, respectively. The tooth was considered to be present if its status was categorized as 0, 1, 3, 6, or 7. Otherwise, the tooth was considered to be absent [33]. The estimated number of teeth, excluding the third molars, could range from 0 to 28 and was categorized into four groups: 0–9, 10–20, 21–26, and ≥27 [34,35,36]. Tooth loss was defined as ≤16 teeth [37]. Chewing ability was rated on a 5-point scale using the following question: “Do you experience discomfort when chewing food due to mouth-related issues, such as teeth, dentures, or gum problems? (If you use dentures, please indicate the discomfort experienced when chewing while wearing them).” Responses of “very uncomfortable” and “uncomfortable” were considered as the presence of chewing difficulty, whereas responses of “moderate”, “not uncomfortable”, and ”not uncomfortable at all” were considered as its absence [38]. In addition, we defined tooth loss/chewing difficulty as present if an individual had at least one of these conditions. 

### 2.4. Dietary Intake 

To assess dietary intake, we used the 1-day 24 h recall method. Trained dietitians investigated food and beverage consumption during the previous day. We determined the intake of energy, carbohydrates, proteins, fats, dietary fibers, vitamin A, thiamin, riboflavin, niacin, vitamin C, calcium, phosphorus, iron, sodium, and potassium. We also calculated the proportion of energy intake from carbohydrates, proteins, and fats. The food items were categorized into 17 groups, namely grains, potatoes, legumes, nuts and seeds, vegetables, mushrooms, seaweeds, fruits, meats, fish and shellfish, eggs, milk and dairy products, beverages, alcohol, oils, sugars, and seasonings. We estimated the intake for individual food groups as well as the total food intake. For nutrients with a defined recommended dietary allowance [39], we calculated the index of nutritional quality (INQ). For proteins, vitamin A, thiamin, riboflavin, niacin, vitamin C, calcium, phosphorous, and iron, we calculated INQs as nutrient intake per 1000 kcal/recommended dietary allowance of nutrient per 1000 kcal [40]. The INQs were determined for the aforementioned nutrients, with INQ < 1.0 indicating insufficient nutritional intake and INQ ≥ 1.0 indicating sufficient nutritional intake [40]. Individuals with a mean INQ < 0.75 were considered to have undernutrition [41,42]. 

### 2.5. Statistical Analyses 

Continuous variables are presented as the mean ± SE. Categorical variables are presented as the percentage (SE) referring to previous studies using KNHANES data [43,44]. Dietary variables are expressed as LSmean ± SE following adjustment for potential confounders. Potential confounders included age, BMI, smoking, drinking, regular walking, daily frequency of tooth brushing, and number of chronic diseases. We included the number of chronic diseases in potential confounders for the following reasons. 84% of older Korean adults have at least one chronic disease [45]. And comorbidity of chronic diseases appears to be related to number of teeth and dietary intake [46,47,48]. We verified whether characteristics of the study participants differ by breastfeeding duration including age, anthropometry, lifestyle, health status, and reproductive factors using a general linear model with Bonferroni’s multiple comparisons test or the chi-square test. To evaluate the associations of breastfeeding duration with tooth loss and chewing difficulty, we performed the chi-square test. Differences in dietary variables according to breastfeeding duration were determined using a general linear model with Bonferroni’s multiple comparisons test after adjustment for all potential confounders. For dietary variables, we also estimated the *p* for trend using the median for each breastfeeding duration category as a continuous variable. Previous study [49] was referred to testing trend for type 2 diabetes risk by assigning median to each breastfeeding duration category and treating this as a continuous variable. We calculated adjusted odds ratios (ORs) and 95% confidence intervals (CIs) for tooth loss across breastfeeding duration using logistic regression analysis. Adjustments were made for the aforementioned potential confounders. *p* for trends were estimated similarly with dietary variables. Likewise, we examined whether breastfeeding duration is related to the risk of chewing difficulty, tooth loss/chewing difficulty, or undernutrition. And we analyzed whether the relationship between tooth loss/chewing difficulty and undernutrition risk is influenced by breastfeeding duration. Adjusted ORs and 95% CIs for undernutrition risk across tooth loss/chewing difficulty categories were obtained, with stratification by two categories of breastfeeding duration (1–36 and ≥37 months). Potential confounders were adjusted as described previously. The influence of the interaction between breastfeeding duration and tooth loss/chewing difficulty on the undernutrition risk was tested. The *p* for interaction was obtained using logistic regression analysis. 

**Figure 2:**
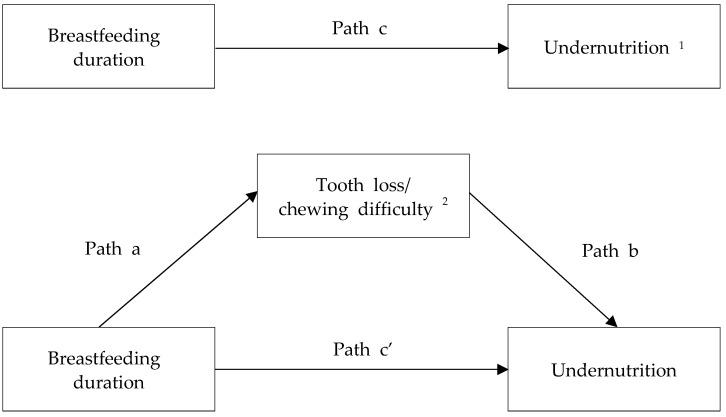
Framework of the mediation analysis by the Baron and Kenny method. ^1^ Mean of proteins, vitamin A, thiamin, riboflavin, niacin, vitamin C, calcium, phosphorous, and iron INQs (index of nutritional qualities) < 0.75; ^2^ at least one of tooth loss (number of teeth ≤ 16) and chewing difficulty (“very uncomfortable” or “uncomfortable” chewing ability). Path c, breastfeeding duration was regressed on undernutrition risk; path a, breastfeeding duration was regressed on tooth loss/chewing difficulty; respective paths c’ and b, breastfeeding duration and tooth loss/chewing difficulty were simultaneously regressed on undernutrition risk.

Furthermore, we assessed the mediating effects of tooth loss and chewing difficulty on the relationship between breastfeeding duration and the undernutrition risk using the Baron and Kenny method. In mediation analysis, breastfeeding duration was divided into 1–36 and ≥37 months. The mediation analysis framework is presented in Figure 2. We conducted four logistic regression analyses after adjusting for potential confounders to determine the beta coefficients, SEs, ORs, and 95% CIs. In the first analysis, breastfeeding duration was regressed on the undernutrition risk (path c). In the second analysis, breastfeeding duration was regressed on the risk of tooth loss/chewing difficulty (path a). The third analysis involved simultaneously regressing breastfeeding duration and tooth loss/chewing difficulty on the undernutrition risk (paths c’ and b, respectively) [50]. A mediating effect was considered present when the following conditions were fulfilled: the association between breastfeeding duration and undernutrition risk was significant (path c); the association between breastfeeding duration and the risk of tooth loss/chewing difficulty was significant (path a); the association between tooth loss/chewing difficulty and undernutrition risk was significant after adjusting for breastfeeding duration (path b); and the association between breastfeeding duration and undernutrition risk (path c) was stronger than the same association after adjusting for tooth loss/chewing difficulty (path c’) [51]. Full mediation was considered to occur if the significance of breastfeeding duration disappeared after adjusting for tooth loss/chewing difficulty (path c’). On the other hand, a partial mediation effect was considered present when breastfeeding duration remained significant after adjusting for tooth loss/chewing difficulty (path c’) [52]. We determined the magnitude of the change in the OR of undernutrition risk explained by tooth loss/chewing difficulty among women who breastfed for ≥37 months compared to those who breastfed for 1–36 months using the following formula: ([OR_path c_ − OR_path c’_]/[OR_path c_ − 1]) × 100 [53]. Furthermore, the Sobel test was conducted to verify the significance of the mediating effect based on the results for paths a and b (http://www.quantpsy.org/sobel/sobel.htm, accessed on 6 November 2023). All statistical analyses were performed using SAS (version 9.4; SAS Institute Inc., Cary, NC, USA).

## 3. Results

### 3.1. Characteristics of Study Participants 

Table 1 presents the characteristics of the study participants. Women who breastfed for 37–72 or ≥73 months were significantly older than those breastfeeding 1–18 or 19–36 months (*p* < 0.001). Those who breastfed for ≥73 months were significantly least likely to walk regularly (*p* < 0.001). And those breastfeeding for 1–18 months were significantly most likely to brush their teeth frequently (*p* = 0.010). Diabetes was significantly more prevalent among women with longer breastfeeding durations (*p* = 0.006). The opposite result was seen for dyslipidemia (*p* = 0.009). Those breastfeeding longer had significantly higher number of pregnancies (*p* < 0.001). 

### 3.2. Number of Teeth and Chewing Ability According to Breastfeeding Duration 

Table 2 presents the number of teeth and chewing ability by breastfeeding duration. There was a significant association between breastfeeding duration and the number of teeth (*p* < 0.001). There was a lower prevalence of 21–26 and ≥27 teeth among women who breastfed for 37–72 or ≥73 months compared to those who breastfed for 1–18 or 19–36 months. The opposite results were observed for 0–9 and 10–20 teeth. The findings for women who breastfed for 1–18 months were similar to those for women who breastfed for 19–36 months. Additionally, women who breastfed for ≥73 months had fewer teeth than those who breastfed for 37–72 months. Prolonged breastfeeding was generally associated with having fewer teeth, particularly when breastfeeding for ≥37 months. 

Chewing ability was also significantly related to breastfeeding duration (*p* = 0.001). Women with longer breastfeeding duration were more likely to report “very uncomfortable” chewing. “Uncomfortable” chewing was slightly more common among women who breastfed for 37–72 or ≥73 months compared to those who breastfed for 1–18 or 19–36 months. The longer the breastfeeding duration, the higher the likelihood of experiencing “uncomfortable” chewing. Women who breastfed for 1–18 months had a higher likelihood of chewing that was “not uncomfortable at all” compared to those who breastfed for longer. In summary, prolonged breastfeeding was associated with poor chewing ability. 

### 3.3. Dietary Intake According to Breastfeeding Duration 

We compared nutrient and food group intake across breastfeeding duration categories (Table 3). Significant differences were observed in the intake of fats, riboflavin, niacin, phosphorus, and potassium, as well as energy intake from carbohydrates and fats, depending on breastfeeding duration. With the exception of energy intake from carbohydrates, reduced intake of these nutrients was significantly associated with longer breastfeeding duration (all *p* < 0.05). Energy intake from carbohydrates was significantly higher among women who breastfed for ≥73 months than among those who breastfed for a shorter duration (*p* < 0.001). The intake of other nutrients did not vary significantly with breastfeeding duration (all *p* > 0.05). Intake of proteins, fats, riboflavin, niacin, vitamin C, calcium, phosphorus, potassium, and energy from proteins and fats displayed a decreasing trend with longer breastfeeding duration (all *p* for trend < 0.05). In contrast, there was a notable upward trend in carbohydrates-derived energy intake with longer breastfeeding duration (*p* for trend < 0.001).

Intake of several food groups significantly varied with breastfeeding duration. Significant negative associations were found for potatoes, eggs, and total food intake with breastfeeding duration (*p* = 0.020, = 0.007, and = 0.002, respectively). Additionally, a significant association was observed for grains (*p* = 0.026). Women who breastfed for ≥73 months showed a significantly higher intake of grains compared to those who breastfed for 19–36 or 37–72 months. No significant differences were noted for the other food groups. Intake of potatoes, nuts and seeds, seaweeds, eggs, milk and dairy products, sugars, and total food tended to decrease with increasing breastfeeding duration (all *p* for trend <0.05). Furthermore, there was an increasing trend in grains intake with longer breastfeeding duration (*p* for trend = 0.010).

We examined whether the INQ is affected by breastfeeding duration (Table S1). There were significant inverse associations of INQ values for proteins, riboflavin, niacin, and phosphorus with breastfeeding duration (*p* = 0.019, = 0.003, = 0.006, and = 0.001, respectively). Women who breastfed for longer tended to have lower INQ for proteins, riboflavin, niacin, calcium, and phosphorus, as well as the mean INQ (all *p* for trend < 0.05). When considering INQ, only women who breastfed for 37–72 and ≥73 months had insufficient protein and vitamin C intake. Insufficient vitamin A intake was observed among all women, except for those who breastfed for 37–72 months. Intake of thiamin, phosphorus, iron, and overall nutrients was sufficient regardless of breastfeeding duration. However, intake of riboflavin, niacin, and calcium was insufficient in all groups. In summary, women who breastfed for longer were more likely to have lower dietary intake for several nutrients and food groups. Conversely, intake of carbohydrates-derived energy and grains appeared to be higher in those breastfeeding for longer periods. 

### 3.4. Risk of Tooth Loss, Chewing Difficulty, and Undernutrition According to Breastfeeding Duration 

**Figure 3:**
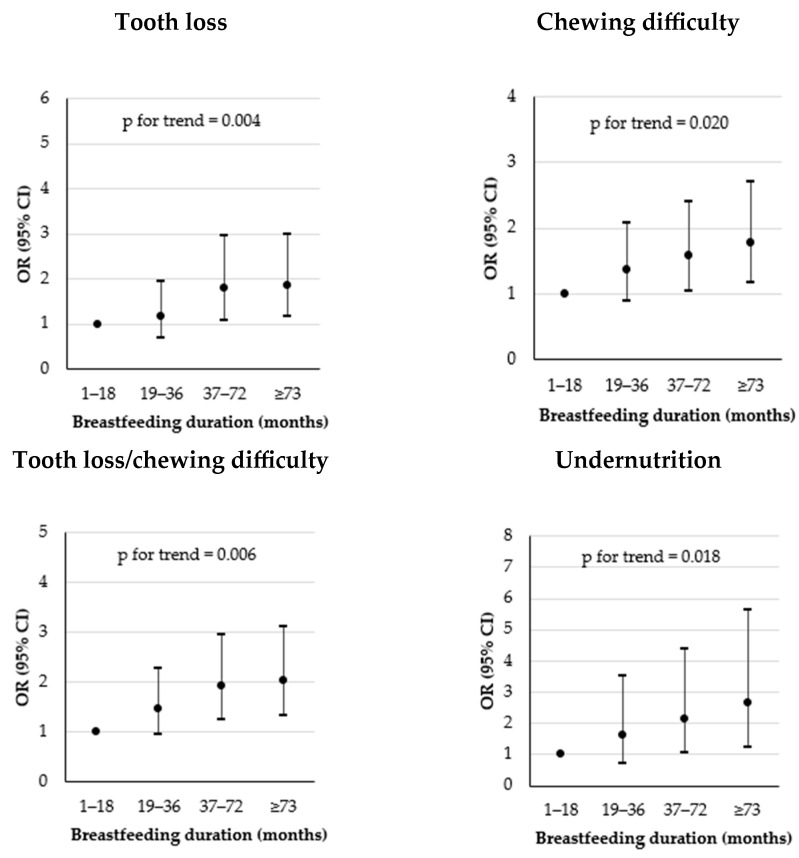
Odds ratio (OR) and 95% confidence interval (95% CI) for tooth loss ^1^, chewing difficulty ^2^, tooth loss/chewing difficulty ^3^, and undernutrition ^4^ according to breastfeeding duration. ^1^ Number of teeth ≤ 16; ^2^ “very uncomfortable” or “uncomfortable” chewing ability; ^3^ at least one of tooth loss (number of teeth ≤ 16) and chewing difficulty (“very uncomfortable” or “uncomfortable” chewing ability); ^4^ mean of proteins, vitamin A, thiamin, riboflavin, niacin, vitamin C, calcium, phosphorous, and iron INQs (index of nutritional qualities) < 0.75. ORs and 95% CIs were calculated using logistic regression analysis after adjustment for age, BMI, smoking, drinking, regular walking, daily frequency of tooth brushing, and number of chronic diseases. *p* for trend were calculated using logistic regression analysis after adjustment for aforementioned potential confounders.

We analyzed the risk of tooth loss, chewing difficulty, tooth loss/chewing difficulty, and undernutrition according to breastfeeding duration (Figure 3). The risk of tooth loss among women who breastfed for 19–36 months was not significantly different from that of women who breastfed for 1–18 months after adjustment for potential confounders (OR = 1.16, 95% CI = 0.69–1.94). However, when compared to those who breastfed for 1–18 months, women who breastfed for 37–72 or ≥73 months were significantly more likely to experience tooth loss (OR = 1.79, 95% CI = 1.08–2.94; OR = 1.86, 95% CI = 1.16–2.97, respectively). Furthermore, as breastfeeding duration increased, there was an increasing trend in the risk of tooth loss (*p* for trend = 0.004). Similar results were observed for chewing difficulty, tooth loss/chewing difficulty, and undernutrition risk.

### 3.5. Risk of Undernutrition According to Tooth Loss/Chewing Difficulty, Stratified by Breastfeeding Duration 

We investigated whether the relationship between tooth loss/chewing difficulty and undernutrition risk varies with breastfeeding duration. Breastfeeding duration was divided into 1–36 and ≥37 months, given that those breastfeeding for 37–72 or ≥73 months were at a higher risk of tooth loss, chewing difficulty, tooth loss/chewing difficulty, and undernutrition. We explored whether there is association between tooth loss/chewing difficulty and undernutrition risk among them. Tooth loss/chewing difficulty was associated with undernutrition risk only among women who breastfed for ≥37 months after adjustment for potential confounders (OR = 1.92, 95% CI = 1.21–3.02) (Table 4). We did not find any interaction effect of tooth loss/chewing difficulty and breastfeeding duration on the risk for undernutrition (*p* = 0.712). 

### 3.6. Mediating Effect of Tooth Loss/Chewing Difficulty on the Association between Breastfeeding Duration and Undernutrition Risk 

Given the above results, we examined the mediating effect of tooth loss/chewing difficulty on the association between breastfeeding duration and undernutrition risk after dividing breastfeeding duration into 1–36 and ≥37 months. Using the Baron and Kenny method, four logistic regression analyses were conducted, adjusting for all potential confounders (Table 5). Compared to women who breastfed for 1–36 months, those who breastfed for ≥37 months had a significantly elevated risk of tooth loss/chewing difficulty (path a) (OR = 1.48, 95% CI = 1.14–1.92) and undernutrition (path c) (OR = 1.63, 95% CI = 1.09–2.46). Women with tooth loss/chewing difficulty had a significantly higher undernutrition risk compared to those without tooth loss/chewing difficulty (path b) (OR = 1.89, 95% CI = 1.29–2.77). After adjusting for tooth loss/chewing difficulty, the association between breastfeeding duration and undernutrition risk was weakened (path c’) (OR = 1.57, 95% CI = 1.04–2.37), although it remained statistically significant, suggesting that this association was partially mediated (9.5%) by tooth loss/chewing difficulty. We conducted the Sobel test to examine whether tooth loss/chewing difficulty has a significant mediating effect on the association between breastfeeding duration and undernutrition risk. The results showed a significant partial mediating effect of tooth loss/chewing difficulty (z = 2.167, *p* = 0.030). 

## 4. Discussion

This study found that breastfeeding duration is positively associated with the risk of tooth loss, chewing difficulty, and undernutrition among older Korean women, particularly those who breastfed for ≥37 months. Among women who breastfed for ≥37 months, the presence of tooth loss or chewing difficulty was associated with a higher undernutrition risk. Furthermore, the association between breastfeeding duration and undernutrition risk was partially mediated by tooth loss and chewing difficulty. 

Our results demonstrated that prolonged breastfeeding is associated with having fewer teeth among older women, particularly if they had breastfed for ≥37 months. These results are in agreement with those of Han et al. [18] as described in Introduction. Recovery of bone loss after weaning is less likely to occur for alveolar bone, which could result in tooth loss among women who breastfed for longer durations. Nevertheless, few studies have investigated this issue. Additional animal and human studies are warranted to assess the association between breastfeeding and tooth loss. The current study demonstrated that older women who breastfed for a prolonged duration, particularly ≥37 months, had poor chewing ability. This relationship might be explained by the association between breastfeeding for ≥37 months and having fewer teeth, as well as the importance of tooth retention for chewing ability [19]. 

Although potential confounders were adjusted, residual confounding might still be present to influence the link between breastfeeding duration and tooth loss risk. Age, regular walking, daily frequency of tooth brushing, and presence of diabetes differed by breastfeeding duration in our study. These variables have been known to influence tooth loss risk as follows: Among older adults in rural Colorado, the risk for losing teeth ≥6 increases with increasing age [54]. The authors interpreted the result could be due to accumulated oral disease for life. A systematic review and meta-analysis of observational studies [55] demonstrated that physical activity is connected to lower prevalence of periodontal disease. Periodontal disease is a chronic inflammatory condition, characterized by alveolar bone loss [56]. Physical activity regulates cytokines including C-reactive protein [57]. Tooth brushing less than twice a day and elevated fasting blood glucose level are related to losing teeth > 8 in general population of Poland [58]. Tooth brushing is required to prevent and not to worsen dental caries and periodontal disease. These conditions are primary causes of tooth loss in adults [59]. And a possible explanation for latter is an inter-relationship between diabetes mellitus and periodontal disease [60].

In this study, women with prolonged breastfeeding had a reduced dietary intake. The breastfeeding duration was negatively associated with the intake of proteins, fats, riboflavin, niacin, vitamin C, calcium, phosphorus, potassium, and energy from proteins and fats. Conversely, it was positively associated with carbohydrates-derived energy intake. Furthermore, women who breastfed for longer periods of time had a lower intake of potatoes, nuts and seeds, seaweeds, eggs, milk and dairy products, sugars, and total food. In contrast, grains intake demonstrated the opposite results. Prolonged breastfeeding was associated with lower intake of several food groups, with textures ranging from soft to hard. Chewing not only breaks down food but also mixes it with saliva to form a bolus for swallowing [19,61]. Therefore, the number of teeth and chewing ability can influence the selection of difficult- and easy-to-cut food, as well as foods that require little or no cutting. It is plausible that women with longer breastfeeding durations have fewer teeth and poor chewing ability, leading to reduced intake for multiple food groups, regardless of their textures, and an overall decline in nutrient intake. Conversely, women with prolonged breastfeeding showed an increased intake of grains, which could be a compensatory mechanism to make up for the lower intake of several food groups. And this might contribute to higher intake of carbohydrates-derived energy among them. Our results are in line with those of previous studies that have reported that individuals with fewer teeth or impaired chewing ability have reduced intake of nutrients and certain food groups, with some exceptions [20,21]. However, nutrients or food groups that exhibit lower or higher intake among individuals with fewer teeth or poor chewing ability have differed between studies. These discrepancies can be attributed to differences among studies in design, populations, the methods used to assess the number of teeth and chewing ability, and the dietary assessment methods. Additionally, we investigated the association between breastfeeding duration and the risk of undernutrition, defined by the INQ. We observed an increasing risk of undernutrition with longer breastfeeding duration, which may be attributed to lower nutrient intake. Women who breastfed for ≥37 months had a higher undernutrition risk compared to those who breastfed for 1–18 months. This may be because the likelihood of experiencing tooth loss and chewing difficulty increased only among those who breastfed for ≥37 months. Chewing is the first step in the digestive process in which food is crushed with the use of teeth to allow easy and safe swallowing [19,61]. And taste, flavor, and texture of food as well as auditory information of crispy products are perceived during chewing and affect food appreciation [62]. Thus, the reduced number of teeth and impaired chewing ability could influence the undernutrition risk due to changed dietary intake. This hypothesis is supported by evidence from previous studies [20,21].

Dietary intake may change after prolonged breastfeeding due to factors other than the number of teeth and chewing ability. In traditional Korean culture, mothers are often advised to avoid eating certain foods during breastfeeding, although there is no scientific evidence to support these recommendations. They commonly avoid consuming caffeine, spicy foods, raw foods, cold foods, and sikhye (a traditional sweet Korean rice beverage) during breastfeeding [63]. Korean often use red pepper powder (a Korean chili-based product) making foods. This product is a major ingredient in Korean spicy foods [64]. Therefore, decrease in overall dietary intake could be caused by spicy foods restriction in Korea. For women who breastfeed for prolonged periods, these modified dietary habits during breastfeeding can persist throughout their lives. It is plausible that prolonged breastfeeding leads to altered dietary intake due to unnecessary self-restriction of certain foods among older Korean women. On the other hand, there could be a bidirectional association between the number of teeth, chewing ability, and nutritional status. While the number of teeth and chewing ability could modify nutritional status [20,21], the reverse may also be true. Based on the INQ, only women who breastfed for ≥37 months had insufficient vitamin C intake. A systematic review by Tada and Miura [65] demonstrated that vitamin C reduces the risk of periodontal disease. Two case–control studies have shown that patients with periodontitis have lower dietary intake and blood levels of vitamin C compared to controls [66,67]. Moreover, two cohort studies demonstrated that individuals with lower dietary intake or blood levels of vitamin C have a higher likelihood of periodontal disease [68,69]. Vitamin C administration improves periodontal parameters in two randomized controlled trials [70,71]. Taken together, it is possible that insufficient vitamin C intake in women engaged in prolonged breastfeeding contributes to their lower number of teeth and worse chewing ability, as they might be at a higher risk for periodontal disease. 

In this study, we examined whether the undernutrition risk is influenced by the presence of tooth loss or chewing difficulty, following stratification by two categories of breastfeeding duration (1–36 and ≥37 months). Tooth loss and chewing difficulty were associated with a higher likelihood of undernutrition in women who breastfed for ≥37 months. Furthermore, we investigated whether the presence of tooth loss or chewing difficulty mediates the relationship between breastfeeding duration and undernutrition risk. We found that tooth loss and chewing difficulty partially mediate the association between breastfeeding duration and undernutrition risk, suggesting that the prevention of tooth loss and chewing difficulty can reduce the risk for breastfeeding-associated undernutrition in older women. Therefore, efforts should be made to maintain good oral health, including having more teeth and ensuring good chewing ability, for older Korean women who breastfeed for ≥37 months to prevent undernutrition. 

The current study had certain limitations. First, data from KNHANES were cross-sectional. Therefore, it is impossible to determine a causal relationship. Second, self-reported breastfeeding duration was recorded, which might have introduced recall bias. Third, chewing ability was evaluated based on participants’ self-reports, which might be less accurate than objective clinical examinations of chewing ability. Fourth, dietary assessment based on a single 24 h recall might not accurately reflect the typical dietary intake. Lastly, our results should be interpreted with some caution. Many of older Korean women are likely to have unusually prolonged breastfeeding duration. Therefore, the association between breastfeeding duration and the risk for tooth loss, chewing difficulty and undernutrition could not be found in other populations. However, this study also had certain strengths. This is the first study to evaluate the association between breastfeeding duration and risk of tooth loss among older women. And no previous study has investigated the relationships between breastfeeding duration with the risk of chewing difficulty and undernutrition. Furthermore, we assessed the mediating effects of tooth loss and chewing difficulty on the association between the breastfeeding duration and undernutrition risk. Further studies are needed to replicate our results. 

## 5. Conclusions

Prolonged breastfeeding is associated with a higher risk of tooth loss, chewing difficulty, and undernutrition among older Korean women, particularly if they breastfed for ≥37 months. Among women who breastfed for ≥37 months, tooth loss and chewing difficulty are related to undernutrition risk. Furthermore, the association between breastfeeding duration and undernutrition risk is mediated by tooth loss and chewing difficulty. Interventions aimed at improving the number of teeth and chewing ability can in turn improve the nutritional status among older Korean women who have breastfed for ≥37 months. 

## Figures and Tables

**Table 1:** Characteristics of study participants according to breastfeeding duration (unweighted *n* = 1666).

	Breastfeeding Duration (Months)				*p*
	1–18	19–36	37–72	≥73	
Unweighted *n*	161	411	559	535	
Age (years)	70.1 ± 0.4 ^c^	70.1 ± 0.3 ^c^	72.5 ± 0.2 ^b^	75.2 ± 0.2 ^a^	<0.001
Height (cm)	151.8 ± 0.5 ^ab^	152.8 ± 0.3 ^a^	151.3 ± 0.3 ^b^	149.9 ± 0.3 ^c^	<0.001
Weight (kg)	55.4 ± 0.7 ^bc^	57.2 ± 0.5 ^a^	56.3 ± 0.4 ^ab^	54.7 ± 0.5 ^c^	0.001
BMI (kg/m^2^)	24.0 ± 0.3	24.4 ± 0.2	24.6 ± 0.1	24.3 ± 0.2	0.199
Smoking (yes)	3.5 (1.7)	1.7 (0.7)	2.6 (0.8)	2.6 (0.7)	0.694
Drinking (yes)	23.3 (3.7)	19.2 (2.4)	20.6 (1.9)	15.2 (1.7)	0.098
Regular walking (yes)	38.7 (4.3)	37.5 (2.8)	34.7 (2.5)	23.1 (2.1)	<0.001
Daily frequency of tooth brushing					
≤1	15.9 (3.3)	12.7 (2.0)	19.3 (2.1)	20.1 (1.9)	0.010
2	38.0 (4.4)	52.1 (2.8)	49.7 (2.5)	47.1 (2.2)	
≥3	46.0 (4.5)	35.2 (2.6)	31.0 (2.4)	32.8 (2.3)	
Hypertension (yes)	48.8 (4.3)	54.6 (2.9)	56.0 (2.3)	60.9 (2.6)	0.079
Diabetes (yes)	13.5 (2.5)	16.6 (2.0)	24.0 (2.1)	22.9 (2.3)	0.006
Dyslipidemia (yes)	39.2 (4.1)	38.3 (2.6)	30.0 (2.3)	28.9 (2.3)	0.009
Stroke/myocardial infarction/ angina pectoris (yes)	15.1 (3.4)	8.3 (1.5)	12.6 (1.6)	15.2 (2.0)	0.045
Cancer (yes)	10.4 (2.6)	7.7 (1.5)	8.1 (1.4)	6.4 (1.3)	0.538
Number of chronic diseases ^1^					
0	27.7 (4.0)	27.8 (2.4)	26.1 (2.1)	23.4 (2.1)	0.847
1	35.0 (4.2)	35.7 (2.7)	35.2 (2.4)	35.7 (2.5)	
≥2	37.3 (4.0)	36.5 (2.6)	38.7 (2.3)	40.9 (2.7)	
Number of pregnancies	4.1 ± 0.2 ^d^	5.0 ± 0.1 ^c^	5.6 ± 0.1 ^b^	6.6 ± 0.1 ^a^	<0.001
Time since last birth (years)	40.6 ± 0.7 ^ab^	41.0 ± 0.3 ^b^	42.0 ± 0.3 ^a^	41.6 ± 0.3 ^ab^	0.031
Breastfeeding duration (months)	10.6 ± 0.4 ^d^	30.6 ± 0.3 ^c^	58.0 ± 0.5 ^b^	123.9 ± 2.2 ^a^	<0.001

Data are expressed as the mean ± SE or percentage (SE). ^1^ The chronic diseases included hypertension, diabetes, dyslipidemia, stroke/myocardial infarction/angina pectoris and cancer. *p* values were calculated using a general linear model or the chi-square test. Different letters indicate significant differences with Bonferroni’s multiple comparisons test (a > b > c > d).

**Table 2:** Number of teeth and chewing ability according to breastfeeding duration.

	Breastfeeding Duration (Months)				*p*
	1–18	19–36	37–72	≥73	
Number of teeth					
0–9	14.5 (3.2)	12.8 (1.9)	24.5 (2.1)	33.8 (2.3)	<0.001
10–20	22.2 (3.6)	24.9 (2.4)	28.1 (2.2)	30.8 (2.2)	
21–26	41.1 (4.5)	40.0 (2.5)	34.2 (2.3)	27.3 (2.3)	
≥27	22.2 (3.5)	22.2 (2.2)	13.2 (1.6)	8.1 (1.2)	
Chewing ability					
Very uncomfortable	7.1 (2.0)	12.6 (1.9)	16.2 (1.9)	22.1 (2.1)	0.001
Uncomfortable	28.5 (4.0)	29.7 (2.6)	31.9 (2.3)	31.5 (2.3)	
Moderate	17.7 (3.2)	20.5 (2.2)	18.3 (1.9)	14.6 (1.7)	
Not uncomfortable	17.9 (3.7)	16.5 (2.2)	12.5 (1.5)	10.8 (1.4)	
Not uncomfortable at all	28.9 (4.0)	20.7 (2.0)	21.1 (1.9)	21.0 (2.0)	

Data are expressed as the percentage (SE). *p* values were calculated using the chi-square test.

**Table 3:** Daily dietary intake according to breastfeeding duration.

	Breastfeeding Duration (Months)				*p*	*p* for Trend
	1–18	19–36	37–72	≥73		
Nutrient						
Energy (kcal)	1592.1 ± 61.8	1536.3 ± 43.7	1508.0 ± 31.9	1550.3 ± 39.0	0.526	0.985
Carbohydrates (g)	286.4 ± 12.4	273.9 ± 8.6	272.7 ± 6.2	289.4 ± 7.9	0.202	0.236
Proteins (g)	51.5 ± 2.2	50.7 ± 1.6	48.3 ± 1.5	47.3 ± 1.4	0.165	0.040
Fats (g)	24.8 ± 1.6 ^a^	24.3 ± 1.2 ^a^	22.0 ± 1.0 ^a^	19.0 ± 1.0 ^b^	<0.001	<0.001
Dietary fibers (g)	23.7 ± 1.3	22.4 ± 0.9	21.6 ± 0.8	21.4 ± 0.8	0.304	0.109
Vitamin A (µg RAE)	520.0 ± 62.2	578.6 ± 50.6	583.5 ± 52.5	543.7 ± 52.3	0.804	0.803
Thiamin (mg)	1.67 ± 0.07	1.54 ± 0.05	1.51 ± 0.04	1.55 ± 0.04	0.246	0.549
Riboflavin (mg)	1.01 ± 0.05 ^ab^	1.05 ± 0.05 ^a^	0.92 ± 0.04 ^bc^	0.84 ± 0.03 ^c^	<0.001	<0.001
Niacin (mg)	12.3 ± 0.6 ^ab^	12.6 ± 0.6 ^a^	11.3 ± 0.4 ^bc^	11.1 ± 0.4 ^c^	0.023	0.006
Vitamin C (mg)	120.3 ± 11.3	107.9 ± 12.6	96.2 ± 6.9	92.1 ± 7.0	0.105	0.039
Calcium (mg)	411.6 ± 24.9	400.1 ± 17.9	387.7 ± 17.9	368.8 ± 16.5	0.273	0.049
Phosphorus (mg)	872.6 ± 38.2 ^a^	832.3 ± 25.8 ^ab^	786.9 ± 23.0 ^bc^	767.6 ± 21.5 ^c^	0.015	0.003
Iron (mg)	13.9 ± 1.0	15.2 ± 0.9	14.3 ± 0.8	14.8 ± 1.2	0.721	0.916
Sodium (mg)	2814.0 ± 163.4	2991.7 ± 122.8	2809.2 ± 108.2	2850.1 ± 112.6	0.611	0.681
Potassium (mg)	2980.9 ± 182.1 ^a^	2581.3 ± 97.7 ^b^	2509.0 ± 76.4 ^b^	2412.8 ± 82.0 ^b^	0.012	0.002
Carbohydrates (%)	73.0 ± 0.9 ^b^	73.1 ± 0.7 ^b^	74.2 ± 0.6 ^b^	76.3 ± 0.6 ^a^	<0.001	<0.001
Proteins (%)	12.9 ± 0.3	13.2 ± 0.2	12.9 ± 0.2	12.5 ± 0.2	0.052	0.009
Fats (%)	14.0 ± 0.7 ^a^	13.7 ± 0.5 ^a^	12.9 ± 0.5 ^a^	11.2 ± 0.5 ^b^	<0.001	<0.001
Food group						
Grains (g)	261.2 ± 13.8 ^ab^	260.8 ± 11.4 ^b^	260.4 ± 8.3 ^b^	294.2 ± 10.1 ^a^	0.026	0.010
Potatoes (g)	79.4 ± 24.2 ^a^	40.5 ± 9.0 ^ab^	41.4 ± 6.8 ^ab^	30.9 ± 6.4 ^b^	0.020	0.005
Legumes (g)	46.3 ± 9.2	36.4 ± 4.0	35.6 ± 3.4	31.5 ± 3.8	0.407	0.094
Nuts and seeds (g)	8.1 ± 2.1	6.9 ± 1.3	5.5 ± 1.1	4.4 ± 0.9	0.206	0.033
Vegetables (g)	342.7 ± 23.1	308.0 ± 14.4	299.7 ± 13.3	305.5 ± 13.4	0.377	0.384
Mushrooms (g)	5.6 ± 1.8	3.5 ± 1.0	4.6 ± 1.5	3.6 ± 1.0	0.645	0.437
Seaweeds (g)	31.0 ± 10.1	40.4 ± 7.2	28.4 ± 6.2	21.1 ± 5.6	0.151	0.037
Fruits (g)	227.2 ± 28.8	207.6 ± 24.9	187.9 ± 15.4	177.0 ± 14.6	0.301	0.065
Meats (g)	51.2 ± 10.7	77.7 ± 12.1	76.1 ± 16.9	50.8 ± 10.4	0.116	0.144
Fish and shellfish (g)	98.2 ± 22.8	97.9 ± 10.5	96.1 ± 12.0	80.6 ± 9.7	0.475	0.142
Eggs (g)	15.9 ± 2.5 ^a^	15.6 ± 1.9 ^a^	14.0 ± 1.9 ^a^	9.2 ± 1.5 ^b^	0.007	0.001
Milk and dairy products (g)	84.2 ± 11.0	72.5 ± 9.7	63.8 ± 6.8	54.2 ± 6.3	0.050	0.007
Beverages (g)	125.3 ± 54.0	57.3 ± 15.8	39.6 ± 7.5	30.8 ± 7.6	0.307	0.069
Alcohol (g)	23.4 ± 7.9	16.0 ± 3.9	17.3 ± 4.6	19.0 ± 4.3	0.715	0.952
Oils (g)	4.2 ± 0.4	4.4 ± 0.4	4.3 ± 0.4	3.8 ± 0.4	0.581	0.213
Sugars (g)	7.1 ± 1.1	7.9 ± 1.0	8.2 ± 0.9	6.0 ± 0.7	0.156	0.045
Seasonings (g)	31.4 ± 3.7	39.5 ± 2.8	35.9 ± 2.4	37.3 ± 2.4	0.225	0.695
Total (g)	1344.8 ± 65.6 ^a^	1232.3 ± 47.8 ^ab^	1153.3 ± 34.1 ^bc^	1119.9 ± 34.0 ^c^	0.002	<0.001

Data are expressed as LSmean ± SE. *p* values were calculated using a general linear model after adjustment for age, BMI, smoking, drinking, regular walking, daily frequency of tooth brushing, and number of chronic diseases. Different letters indicate significant differences with Bonferroni’s multiple comparisons test (a > b > c). *p* for trend were calculated using a general linear model after adjustment for aforementioned potential confounders.

**Table 4:** Odds ratio (OR) and 95% confidence interval (95% CI) for undernutrition ^1^ according to tooth loss/chewing difficulty ^2^, stratified by breastfeeding duration.

Tooth Loss/ Chewing Difficulty	Breastfeeding Duration (Months)				*p* for Interaction
	1–36		≥37		
	OR	95% CI	OR	95% CI	
No	1		1		0.712
Yes	1.74	0.79–3.84	1.92	1.21–3.02	

^1^ Mean of proteins, vitamin A, thiamin, riboflavin, niacin, vitamin C, calcium, phosphorous, and iron INQs (index of nutritional qualities) < 0.75; ^2^ at least one of tooth loss (number of teeth ≤ 16) and chewing difficulty (“very uncomfortable” or “uncomfortable” chewing ability). ORs and 95% CIs were calculated using logistic regression analysis after adjustment for age, BMI, smoking, drinking, regular walking, daily frequency of tooth brushing, and number of chronic diseases. p for interaction were calculated using logistic regression analysis after adjustment for aforementioned potential confounders.

**Table 5:** Mediating effect of tooth loss/chewing difficulty ^1^ on the association between breastfeeding duration ^2^ and undernutrition ^3^ risk.

		Beta	SE	OR	95% CI	*p*	Sobel Test
							Z (*p*)
Path c	Breastfeeding duration → Undernutrition	0.490	0.209	1.63	1.09–2.46	0.019	2.167 (0.030)
Path a	Breastfeeding duration → Tooth loss/chewing difficulty	0.390	0.134	1.48	1.14–1.92	0.004	
Path c’	Breastfeeding duration → Undernutrition	0.448	0.211	1.57	1.04–2.37	0.033	
Path b	Tooth loss/chewing difficulty → Undernutrition	0.636	0.196	1.89	1.29–2.77	0.001	

^1^ At least one of tooth loss (number of teeth ≤ 16) and chewing difficulty (“very uncomfortable” or “uncomfortable” chewing ability); ^2^ breastfeeding for ≥ 37 months vs. 1–36 months; ^3^ mean of proteins, vitamin A, thiamin, riboflavin, niacin, vitamin C, calcium, phosphorous, and iron INQs (index of nutritional qualities) < 0.75. OR, odds ratio; 95% CI, 95% confidence interval. Path c, breastfeeding duration was regressed on undernutrition risk; path a, breastfeeding duration was regressed on tooth loss/chewing difficulty; respective paths c’ and b, breastfeeding duration and tooth loss/chewing difficulty were simultaneously regressed on undernutrition risk. ORs and 95% CIs were calculated using logistic regression analysis after adjustment for age, BMI, smoking, drinking, regular walking, daily frequency of tooth brushing, and number of chronic diseases.

## Data Availability

The data are available from the KNHANES website.

## Supplementary Materials

The following supporting information can be downloaded at: https://www.mdpi.com/article/10.3390/nu15245024/s1, Table S1: Index of nutritional quality (INQ) according to breastfeeding duration.
