# Supplementary material for: Breastfeeding Duration Is Associated with the Risk of Tooth Loss, Chewing Difficulty, and Undernutrition among Older Korean Women: Results of the Korea National Health and Nutrition Examination Survey (KNHANES) 2013–2015
Source: Nutrients. 2023 Dec 7;15(24):5024. doi: 10.3390/nu15245024 (PMC10745866; doi:10.3390/nu15245024)
Supplement: Supplementary file 1 [file nutrients-15-05024-s001.zip › nutrients-2731996-supplementary.pdf]

Table S1. Index of nutritional quality (INQ) according to breastfeeding duration

	Breastfeeding duration (months)				p	p for trend
	1–18	19–36	37–72	≥73		
Proteins	1.00 ± 0.02 [ab]	1.02 ± 0.02 [a]	0.99 ± 0.02 [ab]	0.95 ± 0.01 [b]	0.019	0.002
Vitamin A	0.87 ± 0.12	0.99 ± 0.08	1.02 ± 0.11	0.90 ± 0.08	0.618	0.601
Thiamin	1.78 ± 0.05	1.73 ± 0.03	1.72 ± 0.03	1.72 ± 0.03	0.594	0.421
Riboflavin	0.92 ± 0.04 [ab]	0.99 ± 0.04 [a]	0.88 ± 0.03 [bc]	0.81 ± 0.03 [c]	0.003	< 0.001
Niacin	0.94 ± 0.03 [ab]	0.98 ± 0.03 [a]	0.91 ± 0.02 [bc]	0.88 ± 0.02 [c]	0.006	0.001
Vitamin C	1.13 ± 0.08	1.00 ± 0.09	0.97 ± 0.06	0.91 ± 0.06	0.179	0.097
Calcium	0.51 ± 0.03	0.51 ± 0.02	0.49 ± 0.02	0.46 ± 0.02	0.177	0.039
Phosphorus	1.20 ± 0.03 [ab]	1.21 ± 0.02 [a]	1.16 ± 0.02 [b]	1.11 ± 0.02 [c]	0.001	< 0.001
Iron	1.78 ± 0.06	2.11 ± 0.16	1.91 ± 0.07	1.93 ± 0.09	0.103	0.791
Mean	1.13 ± 0.03	1.17 ± 0.03	1.12 ± 0.02	1.08 ± 0.02	0.101	0.020

Data are expressed as LSmean ± SE. p values were calculated using general linear model after adjustment for age, BMI, smoking, drinking, regular walking, daily frequency of tooth brushing, and number of chronic diseases. Different letters indicate significant differences with Bonferroni's multiple comparisons test (a > b > c). p for trend were calculated using general linear model after adjustment for afore-mentioned potential confounders.
